# Measuring Patient Safety Climate in Acute Stroke Therapy

**DOI:** 10.3389/fneur.2021.686649

**Published:** 2021-10-01

**Authors:** Ferdinand O. Bohmann, Joachim Guenther, Katharina Gruber, Tanja Manser, Helmuth Steinmetz, Waltraud Pfeilschifter

**Affiliations:** ^1^Department of Neurology, University Hospital Frankfurt, Goethe University, Frankfurt am Main, Germany; ^2^FHNW School of Applied Psychology, University of Applied Sciences and Arts Northwestern Switzerland (FHNW), Olten, Switzerland; ^3^Klinikum Lüneburg, Klinik für Neurologie und Klinische Neurophysiologie, Lüneburg, Germany

**Keywords:** critical care, stroke, patient safety, safety attitudes questionnaire, neurology, CRM, acute stroke care, emergency care

## Abstract

**Background:** Treatment of acute stroke is highly time-dependent and performed by a multiprofessional, interdisciplinary team. Interface problems are expectable and issues relevant to patient safety are omnipresent. The Safety Attitudes Questionnaire (SAQ) is a validated and widely used instrument to measure patient safety climate. The objective of this study was to evaluate the SAQ for the first time in the context of acute stroke care.

**Methods:** A survey was carried out during the STREAM trial (NCT 032282) at seven university hospitals in Germany from October 2017 to October 2018. The anonymous survey included 33 questions (5-point Likert scale, 1 = disagree to 5 = agree) and addressed the entire multiprofessional stroke team. Statistical analyses were used to examine psychometric properties as well as descriptive findings.

**Results:** 164 questionnaires were completed yielding a response rate of 66.4%. 67.7% of respondents were physicians and 25.0% were nurses. Confirmatory Factor Analysis revealed that the original 6-factor structure fits the data adequately. The SAQ for acute stroke care showed strong internal consistency (α = 0.88). Exploratory analysis revealed differences in scores on the SAQ dimensions when comparing physicians to nurses and when comparing physicians according to their duration of professional experience.

**Conclusion:** The SAQ is a helpful and well-applicable tool to measure patient safety in acute stroke care. In comparison to other high-risk fields in medicine, patient safety climate in acute stroke care seems to be on a similar level with the potential for further improvements.

**Trial registration:** www.ClinicalTrials.gov Identifier: NCT032282.

## Introduction

Ensuring patient safety has a tremendous value in medicine and is especially demanding in time-critical operations like acute stroke care with critically ill patients and the involvement of interdisciplinary, multiprofessional teams. The fast growing implementation of endovascular therapies in acute stroke care enforces this development and challenges local stroke teams every day. Thus, current guidelines on the management of acute ischemic stroke recommend the establishment of dedicated multidisciplinary stroke teams and the implementation of education programs focusing on team performance and patient safety (1).

In line with safety concepts developed in non-medical high-risk environments, it has been established that patient safety largely depends on human and organizational factors (2–4) and is often challenged at organizational interfaces such as handovers that increase the risk for potential error (5). Safety culture is seen as the basis for ensuring patient safety through successful team performance in emergency medicine (4, 6).

Healthcare professionals' perceptions of safety culture (i.e., patient safety climate) has been shown to correlate with safety outcomes in hospital settings (7–10). Thus, measuring the perceived patient safety climate is important for understanding and effectively addressing patient safety issues. From that future patient safety improvement programs in acute stroke therapy might benefit.

To gauge patient safety climate, the Safety Attitudes Questionnaire (SAQ) has been developed (11). Adopted to various clinical settings and validated in different languages, it is the most widely used instrument for measuring patient safety climate at the team or department level (12). The initial version of the SAQ has 60 items, including 34 core items, which are independent of the clinical setting. The short version of SAQ only includes the core items. Psychometric properties data from the SAQ identified six factors for safety culture: teamwork climate, job satisfaction, safety climate, stress recognition, perception of management and working conditions (Table 1). For intensive care units (ICU), the SAQ factors have already proven to be sensitive for changes by a quality improvement program, associated with reductions in medication errors and with shorter lengths of stay (13). It has been also shown that critical care units with highest scores on SAQ factors had the lowest rates of blood-stream infections (11, 14). Based on real-life studies targeting safety climate (7–10), the proposed cut-off for each SAQ factor should be 60 point (on a 100 point scale), respectively, 3.4 points on the 5-point Likert scale (5, 11).

**Table 1:** SAQ factors, items and dimensions.

**SAQ factors, items and dimensions**	
	**Teamwork climate (6 items)**
1	Nurse input is well received in this clinical area.
2	In this clinical area, it is difficult to speak up if I perceive a problem with patient care.
3	Disagreements in this clinical area are resolved appropriately (i.e., not who is right, but what is best for the patient)
4	I have the support I need from other personnel to care for patients.
5	It is easy for personnel in this clinical area to ask questions when there is something that they do not understand.
6	The physicians and nurses here work together as a well-coordinated team.
	**Safety climate (7 items)**
7	I would feel safe being treated here as a patient.
8	Medical errors are handled appropriately in this clinical area.
9	I know the proper channels to direct questions regarding patient safety in this clinical area.
10	I receive appropriate feedback about my performance.
11	In this clinical area, it is difficult to discuss errors.
12	I am encouraged by my colleagues to report any patient safety concerns I may have.
13	The culture in this clinical area makes it easy to learn from the errors of others.
	**Job satisfaction (5 items)**
14	I like my job.
15	Working in this hospital is like being part of a large family.
16	This hospital is a good place to work.
17	I am proud to work at this hospital.
18	Moral in this clinical area is high.
	**Stress recognition (4 items)**
19	When my workload becomes excessive, my performance is impaired.
20	I am less effective at work when fatigued.
21	I am more likely to make errors in tense or hostile situations.
22	Fatigue impairs my performance during emergency situations (e.g., emergency resuscitation, seizure).
	**Perception of Management (8 items)**
	*Unit level:*
23	Management supports my daily efforts.
24	Management does not knowingly compromise the safety of patients.
25	Problem personnel are dealt constructively in hospital.
26	I am provided with adequate, timely information about events in the hospital that might affect my work.
	*Hospital level:*
27	Management supports my daily efforts:
28	Management does not knowingly compromise the safety of patients.
29	Problem personnel are dealt constructively in hospital.
30	I am provided with adequate, timely information about events in the hospital that might affect my work.
	**Working conditions (4 items)**
31	The levels of staffing in this clinical area are sufficient to handle the number of patients.
32	This hospital does a good job of training new personnel.
33	*All the necessary information for diagnostic and therapeutic decisions is routinely available to me (excluded)*.
34	Trainees in my discipline are adequately supervised.

Based on these results, the SAQ might be a valuable tool for assessing patient safety climate in acute stroke therapy. Providing measurable positive effects on patient safety climate might facilitate the long-term organizational anchoring of quality improvement programs. Thus, the aim of this study was to evaluate the SAQ in the setting of acute stroke therapy. To the best of our knowledge, this is the first study using SAQ in the context of clinical neurology.

## Methods

### Design and Setting

From October 1st 2017 to July 1st 2018 a cross sectional survey was conducted at seven stroke centers of tertiary care university hospitals with 24/7 capacity for thrombectomy (University Hospital Augsburg, University Hospital Tuebingen, University Hospital Heidelberg, Ludwig Maximilians-University Munich, Centre for Stroke Research Berlin Charité, University Medical Centre Hamburg, University Hospital Cologne) as part of the Simulation STREAM trial (NCT 032282). The trial was coordinated by the University Hospital Frankfurt (Goethe University) and had the approval of the ethics committee of Frankfurt University Hospital (ID 433/16) with secondary approvals from the ethics committees of all participating centers. The trial intervention itself did not require individual consent.

### Safety Attitudes Questionnaire – German Version

The SAQ was first developed by Sexton and colleagues (11). Zimmermann et al. translated and validated the short version of the SAQ into the German language version (15). Items and dimensions are illustrated in Table 1. By decision of an interdisciplinary expert group, item 33 of the SAQ was not applicable to acute care of stroke patients and excluded before the start of the trial. Answers to the 33 SAQ items are given on a 5-point Likert scale (1 = disagree strongly, 2 = disagree slightly, 3 = neutral, 4 = agree slightly, 5 = agree strongly).

### Data Collection

In each participating center, all members of the stroke teams (professionals involved in acute stroke care: neurologists, neuroradiologists/-interventionalists, nurses, medical technical assistants) received an invitation and two e-mail reminders to fill out the German version of SAQ in a paper and pencil version. Questionnaires (*n* = 247) were administered by a local principle investigator (PI), collected and sent back to the sponsor (University Hospital Frankfurt) for central data collection and analysis.

### Statistical Analysis

#### Psychometric Testing

Factor scale scores were calculated for individual respondents by the taking the average of the specific items per factor. For reliability analysis, Cronbach's alpha was calculated to assess the internal consistency of the overall SAQ. Cronbach's alpha was calculated for each factor of the SAQ (>0.7 indicates adequate internal consistency (16). Separately, scale reliability analysis for each item and dimension resulted in a corrected item-total correlation and Cronbach's alpha. Inter-item correlations were examined for internal consistency reliability of the questionnaire.

Based on the identified factor structure during the testing of the validated original SAQ version and the German translation, a confirmatory factor analysis (CFA) was performed to verify the factor structure in context of acute stroke care (11, 15). CFA based on participants who fully completed the instrument (*n* = 151) with analysis of moment structures (AMOS 26.0.0, IBM, Chicago, USA) software. A Root Mean Square Error of Approximation (RMSEA) <0.08, a Tucker-Lewis Index (TLI) close to 0.95 and a Comparative Fit Index/CFI) > 0.9 (17) are deemed for a successful model (18). Additionally χ^2^ statistics are given (19). Modification indices (MI) were examined to identify any additional adjustments. Factor loadings of individual items were estimated based on the six-factor CFA model.

#### Descriptive Statistics

Frequency tables were used to analyse data and missing values (MV). Scores were reversed for all negatively worded items. Despite the ordinal scaling of SAQ data, the established method is to present results as mean values or percentages (agree/disagree) (9, 20). Screening for outliers and normal distribution was done with boxplots and q-q plots. To illustrate percentages of participants that agreed or disagreed with each specific item on the 5-point Likert scale, values of 1 and 2 were recoded as ‘disagree', 3 as ‘neutral' and 4 and 5 as ‘agree'. A threshold score of 3.4 points on the 5-point Likert scale (representing 60% agreement on the 0–100-point scale where disagree strongly becomes 0, disagree slightly becomes 25, neutral becomes 50, agree slightly becomes 75 and agree strongly becomes 100) should be exceeded, with a “goal zone” of 4.2–5 points (5).

For interpretation of group differences, multivariate analysis of variance (MANOVA) was used to analyse mean scores. Three separate MANOVA's (Wilks Lambda) were performed with professional position, department and work experience of physicians ( ≤ 5 vs. >5 years for medical doctors, 5 years as cut-off for separation resident/ specialist) as independent variables. *post-hoc* univariate ANOVAs were conducted for every dependent variable. Additionally, Tukey HSD *post-hoc* analysis explored differences between two groups. For the correlation analysis of relations between SAQ dimensions, Pearson's correlation was used with a two-tailed test of significance. A *p* < 0.05 was deemed to indicate significance. Data was analyzed with SPSS 26 (IBM; Armonk, BY, USA).

## Results

### Study Sample and Descriptive Statistics

In total 164 questionnaires were returned by participants representing an overall response rate of 66.4%. The complete data set consisted of 111 physicians, 41 nurses and 10 medical technical assistants with regular patient contact (Table 2).

**Table 2:** Participant characteristics.

	**In total** ** (*n* = 164)**	**Physicians (*****n*** **=** **111)**			**Nurses** ** (*n* = 41)**	**MTA^**†**^** ** (*n* = 10)**	**Neurology** ** (*n* = 111)**	**Neuroradiology** **(*n* =35)**
		**In total** ** (*n* = 111)**	**≤5 years^**‡**^** ** (*n* = 60)**	**>5 years^**‡**^** ** (*n* = 50)**				
Age (years), median (IQR)	33 (29–39)	33 (30–37)	30 (29–31)	39 (35–42)	33 (28–44)	30 (27–46)	31 (29–36)	35 (31–43)
Female, n (%)	86 (52.5)	44 (4)	32 (53.3)	11 (22.0)	32 (78.0)	8 (80.0)	61 (55.5)	12 (36.4)
Duration of professional experience (years), median (IQR)								
*- as physician/ nurse*	7 (3-13)	5 (3-10)	3 (2-4)	10 (8-15)	10 (4-23)	8 (3-20)	5 (2-19)	9 (4-18)
*- in acute stroke care*	4 (2-10)	4 (2-8)	2 (1-3)	8 (6-13)	6 (2-10)	4 (2-14)	4 (1-9)	5 (3-10)

† *MTA, Medical technical assistant*;

‡ *Duration of professional experience (years) as physician*.

### SAQ Factor Structure and Reliability

Confirmatory factor analysis based on the retained 33 items with six factors showed good model fit (RMSEA = 0.044, 90% CI 0.032, 0.056; TLI = 0.94, CFI = 0.95, $\chi_{\left(376\right)}^{2}$ = 486.74, *p* < 0.001) (21). Item loadings on the respective factor are presented in Supplementary Table 2.

The internal consistency of the questionnaire is satisfactory, with Cronbach's alpha 0.88. Cronbach's alpha for all factors was above 0.7 (0.73–0.85), except for the factor “perception of management” where Cronbach's alpha was 0.22 (Table 3) indicating heterogeneity in relation to the confidence in adequate institutional management.

**Table 3:** SAQ factors correlations and Cronbach's alpha.

	**Teamwork climate**	**Safety climate**	**Job satisfaction**	**Stress recognition**	**Perception of management**	**Working conditions**
**Correlation (Pearson) and Cronbach's alpha (alpha** **=** **0.88)**						
Teamwork climate	0.748					
Safety climate	0.734	0.816				
Job satisfaction	0.629	0.552	0.824			
Stress recognition	−0.002	−0.052	−0.014	0.845		
Perception of management	0.12	0.294	0.085	0.032	0.215	
Working conditions	0.499	0.593	0.499	−0.142	0.238	0.729

*Cronbach's alpha is highlighted on the diagonal*.

### SAQ Response Pattern

**Figure 1:**
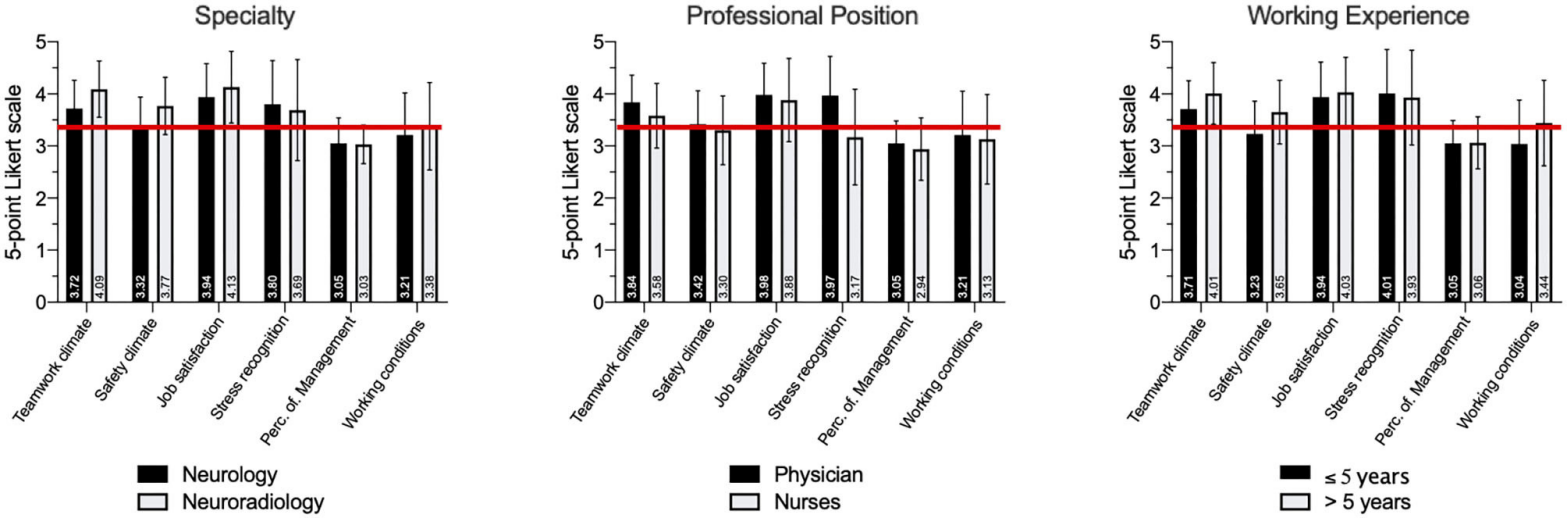
Perceptions of patient safety climate depending on specialty, professional position and duration of professional experience. The six SAQ factors with mean values and standard deviation (SD) are depicted for each subgroup. Individual mean values are written vertical per column. The red lines reflect the proposed benchmark of 3.4 points. Perc. of Management: Perception of Management.

Missing values did not exceed 2.5% (range 0–2.4%). We found no statistical significant difference for MV rates between trial centers, departments or professions. Item 24 (unit level) and 28 (hospital level) presented a bimodal response pattern (Supplementary Table 1). *Post-hoc* feedback concerning item 24 and 28 suggest that these items were not clear to participants. Negative Item-Total-Correlation enforced these findings, so these items were excluded from individual factor analysis. Demographics are presented in Table 3. Mean values and SD for individual SAQ factors are depicted in Figure 1.

### Differences in Patient Safety Climate Across Departments

Comparing the results for the respective SAQ factors, we generally found higher scores for neuroradiology than for neurology or other departments (e.g., anesthetics, neurosurgery). A one-way MANOVA showed a statistically significant difference between departments (neurology, neuroradiology, others) on the combined dependent variables, *F*_(12, 306)_ = 3.327, *p* < 0.001, partial η^2^ = 0.115, Wilk's Λ = 0.782.

*Post-hoc* univariate ANOVAs show a statistically significant difference between the departments for teamwork climate, *F*_(2, 158)_ = 8.049, *p* < 0.001, partial η^2^ = 0.092, safety climate, *F*_(2, 158)_ = 7.866, *p* = 0.001, partial η^2^ = 0.091 and working condition *F*_(2, 158)_ = 2.193, *p* = 0.044, partial η^2^ = 0.039, but not for job satisfaction *F*_(2, 158)_ = 2.808, *p* = 0.195, partial η^2^ = 0.034, stress recognition, *F*_(2, 158)_ = 1.654, *p* = 0.195, partial η^2^ = 0.021, and perception of management *F*_(2, 158)_ = 2.675, *p* = 0.072, partial η^2^ = 0.033 (Table 4).

**Table 4:** Perceptions of patient safety climate per specialty, professional position and working experience.

	**Teamwork climate**	**Safety climate**	**Job satisfaction**	**Stress recognition**	**Perception of management**	**Working conditions**
**Specialty**						
Neurology (*n* =111)	3.72^*^ (0.54)	3.32^*^ (0.62)	3.94 (0.62)	3.80 (0.84)	3.05 (0.49)	3.21^*^ (0.81)
Neuroradiology (*n* = 35)	4.09^*^ (0.54)	3.77^*^ (0.55)	4.13 (0.69)	3.69 (0.97)	3.03 (0.37)	3.38^*^ (0.84)
Others (*n* = 16)	3.54 (0.65)	3.23 (0.69)	3.68 (0.63)	3.38 (0.80)	2.76 (0.62)	2.75 (0.84)
**Professional position**
Physician (*n* = 111)	3.84^*^ (0.52)	3.42 (0.64)	3.98 (0.61)	3.97^*^ (0.75)	3.05 (0.43)	3.21 (0.84)
Nurse (*n* = 41)	3.58^*^ (0.62)	3.30 (0.66)	3.88 (0.80)	3.17^*^ (0.92)	2.94 (0.60)	3.13 (0.86)
MTA (*n* = 10)	4.05^*^ (0.54)	3.73 (0.51)	4.10 (0.68)	3.48^*^ (0.90)	2.99 (0.47)	3.37 (0.88)
**Physician's working experience**
≤ 5 years (*n* = 60)	3.71^*^ (0.54)	3.23^*^ (0.63)	3.94 (0.67)	4.01 (0.84)	3.05 (0.44)	3.04^*^ (0.84)
>5 years (*n* = 50)	4.01^*^ (0.59)	3.65^*^ (0.61)	4.03 (0.67)	3.93 (0.91)	3.06 (0.50)	3.44^*^ (0.82)
**Overall** (*n* = 164)	**3.77 (0.57)**	**3.40 (0.64)**	**3.95 (0.67)**	**3.74 (0.88)**	**3.01 (0.47)**	**3.19 (0.85)**

* *Between groups difference significant p < 0.05 (post-hoc univariate ANOVA respectively Tukey-HSD)*.

Additional Tukey HSD *post-hoc* analysis on *teamwork climate* revealed a significant difference between neurology and neuroradiology, *p* = 0.001 (*M*_Diff_ = −0.3802, 95%–CI[−0.6313, −0.1291]), and between neuroradiology and others, *p* = 0.003 (*M*_Diff_ = 0.5379, 95%–CI[.1583, 0.9393]), but not between neurology and others, *p* = 0.483 (*M*_Diff_ = 0.1686, 95%–CI[−0.1776, 0.5148]).

Tukey HSD *post-hoc* analysis on *safety climate* revealed a significant difference between neurology and neuroradiology, *p* = 0.001 (*M*_Diff_ = −0.4460, 95%–CI[−0.7262, −0.1659]), and between neuroradiology and other, *p* = 0.011 (*M*_Diff_ = 0.5379, 95%–CI[.1023, 0.9736]), but not between neurology and others, *p* = 0.840 (*M*_Diff_ = 0.0919, 95%–CI[−0.2944, 0.4781]).

Tukey HSD *post-hoc* analysis on *working conditions* revealed a significant difference between neuroradiology and other, *p* = 0.034 (*M*_Diff_ = 0.25046, 95%–CI[.0384, 1.2235]) but not between neurology and neuroradiology, *p* = 0.513 (*M*_Diff_ = −0.1779, 95%–CI[−0.5590, 2032]), and between neurology and others, *p* = 0.106 (*M*_Diff_ = 0.4530, 95%–CI[−0.0724, 0.9784]).

### Differences in Patient Safety Climate Across Professions

While teamwork climate was scored higher by physicians and medical technical assistants than by nurses, patient safety, working conditions and job satisfaction did not differ significantly between profession. A one-way MANOVA showed a statistically significant difference between professions on the combined dependent variables, *F*_(18, 433)_ = 3.393, *p* < 0.001, partial η^2^ = 0.117, Wilk's Λ = 0.689. *post-hoc* univariate ANOVAs showed a statistically significant difference between the professions for teamwork climate, *F*_(3, 158)_ = 6.502, *p* < 0.001, partial η^2^ = 0.110 and stress recognition, *F*_(3, 158)_ = 11.056, *p* < 0.001, partial η^2^ = 0.174, but not for safety climate, *F*_(3, 158)_ = 1.652, *p* = 0.180, partial η^2^ = 0.030, job satisfaction *F*_(3, 158)_ = 1.094, *p* = 0.354, partial η^2^ = 0.020, perception of management *F*_(3, 158)_ = 0.548, *p* = 0.548, partial η^2^ = 0.010 and working conditions *F*_(3, 158)_ = 0.877, *p* = 0.454, partial η^2^ = 0.016.

Additionally Tukey HSD *post-hoc* analysis on *teamwork climate* revealed a significant difference between physicians and nurses, *p* = 0.032 (*M*_Diff_ = 0.2543, 95%–CI[.0171, 0.4915]), and between nurses and medical technical assistants, *p* = 0.041 (*M*_Diff_ = −0.4728, 95%–CI[−0.9300, −0.0155]), but not between physicians and medical technical assistants, *p* = 0.451 (*M*_Diff_ = −0.2185, 95%–CI[−0.6467, 0.2097]). Tukey HSD *post-hoc* analysis on *stress recognition* revealed a significant difference between physicians and nurses, *p* < 0.001 (*M*_Diff_ = 0.8025, 95%–CI[.453, 0.1.1515]), but not between nurses and medical technical assistants, *p* = 0.529 (*M*_Diff_ = −0.3063, 95%–CI[−0.9789, 0.3663]) and between physicians and medical technical assistants, *p* = 0.153 (*M*_Diff_ = 0.4962, 95%–CI[−0.1337, 1.1261]).

### Differences in Patient Safety Climate According to the Duration of Professional Experience

Concerning all individual SAQ factors, experienced physicians only scored higher for teamwork climate and working conditions than physicians with less working experience (<5 years). A one-way MANOVA showed a statistically significant influence of the duration of professional experience (physicians with more or <5 years working experience) on the combined dependent variables, *F*_(6, 102)_ = 3.350, *p* = 0.005, partial η^2^ = 0.165, Wilk's Λ = 0.835.

*Post-hoc* univariate ANOVAs showed a statistically significant difference between the levels of experience for teamwork climate, *F*_(1, 107)_ = 2.745, *p* = 0.001, partial η^2^ = 0.095, safety climate, *F*_(1, 107)_ = 4.794, *p* < 0.001, partial η^2^ = 0.109 and working conditions *F*_(1, 107)_ = 4.588, *p* = 0.009, partial η^2^ = 0.062, but not for job satisfaction, *F*_(1, 107)_ = 0.580, *p* = 0.448, partial η^2^ = 0.005, stress recognition *F*_(1, 107)_ = 0.335, *p* = 0.564, partial η^2^ = 0.003 and perception of management *F*_(1, 107)_ = 0.038, *p* = 0.847, partial η^2^ = 0.000.

## Discussion

The increasing implementation of endovascular therapies requires fast interdisciplinary decision-making and the involvement of neurointerventionalists, neurointensive care specialists and anesthetists. Consequences are larger team sizes and an increased number of handovers. Therefore, a good teamwork climate is essential for patient safety. This study explored for the first time the SAQ as a potential assessment tool for safety culture in acute stroke care. The results showed a good reliability and CFA confirmed the proposed factor model for this survey (11). In comparison to benchmarking data from emergency departments and intensive care units from other disciplines than neurology, our results indicate comparable results for teamwork climate and patient safety in the field of acute stroke care with the potential for future refinements (7–10). Noteworthy are particularly high scores for job satisfaction. Our results indicate that the SAQ has the potential to validly depict changes of the safety climate induced by dedicated improvement programs targeting patient safety in acute stroke care.

For quantitative analysis of hospitals' safety climate, several measurement methods have been developed, the most frequently used are the Hospital Survey on Patient Safety Culture (HSPSC), the Safety Organizing Scale (SOS) and the SAQ (11, 22). We chose the SAQ because of its well-characterized psychometric properties, available benchmarking data and verification of the original factor analysis (10, 11, 23). One strength of the SAQ is the possibility to differentiate between different factors of patient safety climate (15, 24). Nevertheless, additional qualitative safety climate measurements, like structured interviews, could be necessary to explore causality of findings (10).

The mean values for the perception of safety climate in the present study were similar to former SAQ studies targeting safety climate at intensive care units or emergency rooms [Table 4, (25)]. Referring to benchmarking data from Sexton and colleagues comparing results of six SAQ versions from different departments and sites (ICU-UK, ICU-NZ, ICU-USA, inpatient-USA, OR-UK, ambulatory-USA) teamwork climate (factor means from the six SAQ versions (range) 3.57–3.97 vs. actual SAQ overall mean 3.77), safety climate (means 3.42–3.80 vs. actual overall mean 3.4), stress recognition (means 3.19–3.98 vs. actual overall mean 3.74), perception of management (means 2.53–3.21 vs. actual overall mean 3.01) and working conditions (means 2.97–3.46 vs. actual overall mean 3.19) were on a similar level. Only job satisfaction scored higher in the present study than in the afore mentioned studies (means 3.38–3.82 vs. actual overall mean 3.95). To the best of our knowledge, this is the first study benchmarking safety climate in acute stroke care against existing data from other clinical areas (11, 12).

In comparison to other studies targeting patient safety climate (7–10), the present study reached the proposed threshold of 3.4 points for the factors teamwork climate, safety climate and stress recognition (5, 11). Nevertheless, the cut-off was not achieved for the factors perception of management and working conditions. Both factors are strongly influenced by hospital management setting the local frame for work and communication. Because of the low internal consistency of the factor “perception of management” (Cronbach's alpha 0.22) further interpretations should be done carefully. A possible explanation for the low internal consistency could be the involvement of different management entities due to the multiprofessional and interdisciplinary composition of the stroke team.

Concerning results for individual SAQ factors in our explorative analysis, physicians scored higher than nurses in most items, especially in items concerning teamwork climate (3.84 ± 0.52 vs. 3,58 ± 0.62, *p* = 0.032) and stress recognition (3.97 ± 0.75 vs. 3.17 ± 0.92, *p* < 0.001), where higher scores indicate a better sensitivity for the impact of stress (Figure 1). Similar results were found elsewhere (25, 26). These might indicate different perceptions of teamwork and stress identify nurses as a particularly vulnerable group. This should be taken into account during team trainings.

Concerning the influence of the duration of professional experience on perceived safety climate, our data suggest that for physicians, a working experience of more than 5 years results in significantly higher scores for teamwork climate (>5 years: 4.01 ± 0.59 vs. ≤ 5 years: 3.71 ± 0.54, *p* = 0.001), safety climate (3.65 ± 0.61 vs. 3.23 ± 0.63, *p* < 0.001) and working conditions (3.44 ± 0.82 vs. 3.04 ± 0.84, *p* = 0.009). This cut-off was chosen because 5 years is the duration of specialty training for neurology in Germany. The achievement of specialist status often confers more work autonomy and a relief from procedural tasks, resulting in more satisfaction as reported elsewhere (20). Interestingly, job satisfaction and stress recognition were independent from working experience with job satisfaction being particularly high in acute stroke care as compared to published results from other clinical environments. When looking at the speciality, we found differences in teamwork climate, safety climate and working conditions with higher scores for neuroradiology. Since the number of respondents and their baseline parameters are significantly different, these findings should be interpreted with caution.

We acknowledge that there are some limitations that we could not circumvent when designing this study: First, we recruited only experienced high-volume stroke centers. Therefore, our findings might not be representative for stroke units in general and a potential selection bias should be considered although we addressed this issue at least partially by employing a multicenter approach. Second, the overall response rate of 66.4% equalled that of previous studies based on the SAQ and was deemed acceptable (11). In studies with voluntary participation, as in the present study, the response rate plays a major role regarding representative statements. This should be considered in future studies to circumvent a possible selection bias. Third, sample size for confirmatory factor analysis was limited due to study design and number of study centers, but results were similar to former factor analysis (25). Fourth, psychometric properties of the SAQ factor perception of management showed lower values than benchmarking data, but patterns were similar (15). Therefore, interpretation of this factor should be done with caution. Despite these restrictions, psychometric properties from similar studies using the SAQ demonstrated good model validity and reliability (15, 25). In principle, the SAQ cannot exclude recall bias, since it asks for a self-assessment. This aspect must be taken into account when assessing the results.

## Conclusions

The German SAQ is a reliable instrument to measure safety climate of stroke services. We found comparatively high rates for job satisfaction among all professions of the stroke team but also indicators for a higher vulnerability of nurses and physicians with <5 years work experience toward unfavorable teamwork climate and working conditions. Further studies are needed to evaluate the potential of interventional studies for improving patient safety climate in stroke medicine and neurocritical care.

## Data Availability Statement

The data that support the findings of this study are available on request from the corresponding author. The data are not publicly available due to privacy or ethical restrictions.

## Ethics Statement

The trial was coordinated by the University Hospital Frankfurt (Goethe University) and had the approval of the ethics committee of Frankfurt University Hospital (ID 433/16) with secondary approvals from the ethics committees of all participating centers. The trial intervention itself did not require individual consent.

## Author Contributions

FB, JG, and WP had full access to all of the data in the study and take responsibility for the integrity of the data and the accuracy of the data analysis, concept and design, and statistical analysis. FB and JG acquisition, analysis, or interpretation of data. FB and WP drafting of the manuscript. FB, JG, KG, TM, HS, and WP critical revision of the manuscript for important intellectual content. FB, WP, and HS supervision. All authors contributed to the article and approved the submitted version.

## Funding

The study was funded by Stryker Neurovascular (grant to WP). The funding source was not involved in study design, monitoring, data collection, statistical analyses, interpretation of results, or manuscript writing.

## Conflict of Interest

The authors declare that the research was conducted in the absence of any commercial or financial relationships that could be construed as a potential conflict of interest.

## Publisher's Note

All claims expressed in this article are solely those of the authors and do not necessarily represent those of their affiliated organizations, or those of the publisher, the editors and the reviewers. Any product that may be evaluated in this article, or claim that may be made by its manufacturer, is not guaranteed or endorsed by the publisher.

## Supplementary Material

Supplementary Table 1 STREAM collaborators.

Supplementary Table 2 Items response rate, means, reliability characteristics and factor loading.
