# Supplementary material for: Measuring Patient Safety Climate in Acute Stroke Therapy
Source: Front Neurol. 2021 Oct 1;12:686649. doi: 10.3389/fneur.2021.686649 (PMC8517443; doi:10.3389/fneur.2021.686649)
Supplement: Supplementary Table 1 — STREAM collaborators. [file Table_1.pdf]

Measuring patient safety climate in acute stroke therapy

Ferdinand O. Bohmann,[1] Joachim Guenther,[1] Katharina Gruber,[1] Tanja Manser,[2] Helmuth Steinmetz,[1] Waltraud Pfeilschifter[1,3] for the STREAM Trial investigators

1- University Hospital Frankfurt, Goethe University, Department of Neurology, Frankfurt am Main, Germany
2- University of Applied Sciences and Arts Northwestern Switzerland (FHNW), FHNW School of Applied Psychology, Olten, Switzerland
3- Klinikum Lüneburg, Klinik für Neurologie und Klinische Neurophysiologie, Lüneburg, Germany

STREAM Collaborators

Name	Location	Role	Contribution
Peter Rostek	University Hospital Frankfurt, NICU Nursing Staff, Frankfurt am Main, Germany	Site Investigator	Acquisition of data
Heike Rai, B.A.	University Hospital Frankfurt, Department of Neurology, Frankfurt am Main, Germany	Site Investigator	Acquisition of data
Philipp Zickler, MD	University Hospital Augsburg, Department of Neurology and Clinical Neurophysiology, Augsburg, Germany	Site Investigator	Acquisition of data
Michael Ertl, MD	University Hospital Augsburg, Department of Neurology and Clinical Neurophysiology, Augsburg, Germany	Site Investigator	Acquisition of data
Ansgar Berlis, MD	University Hospital Augsburg, Department for Diagnostic and Interventional Radiology and Neuroradiology, Augsburg, Germany	Site Investigator	Acquisition of data
Sven Poli, MD	Hertie Institute for Clinical Brain Research, University of Tübingen, Germany	Site Investigator	Acquisition of data
Annerose Mengel, MD	Hertie Institute for Clinical Brain Research, University of Tübingen, Germany	Site Investigator	Acquisition of data
Peter Ringleb, MD	University Hospital Heidelberg, Department of Neurology, Heidelberg, Germany	Site Investigator	Acquisition of data
Simon Nagel, MD	University Hospital Heidelberg, Department of Neurology, Heidelberg, Germany	Site Investigator	Acquisition of data
Johannes Pfaff, MD	University Hospital Heidelberg, Department of Neuroradiology, Heidelberg, Germany	Site Investigator	Acquisition of data
Frank A. Wollenweber, MD	Helios-HSK Wiesbaden, Department of Neurology, Wiesbaden, Germany	Site Investigator	Acquisition of data
Lars Kellert, MD	Ludwig Maximilians-University, Department of Neurology, Munich, Germany	Site Investigator	Acquisition of data
Moriz Herzberg, MD	Universitätsklinikum Würzburg, Department of Diagnostic and Interventional Radiology, Germany	Site Investigator	Acquisition of data
Luzie Köhler, MD	Center for Stroke Research Berlin, Charité - Universitätsmedizin Berlin, Germany	Site Investigator	Acquisition of data
Karl Georg Haeusler, MD	Universitätsklinikum Würzburg, Department of Neurology, Germany	Site Investigator	Acquisition of data
Anna Alegiani, MD	University Medical Center Hamburg Eppendorf, Department of Neurology, Hamburg, Germany	Site Investigator	Acquisition of data
Charlotte Schubert, MD	University Medical Center Hamburg Eppendorf, Department of Neurology, Hamburg, Germany	Site Investigator	Acquisition of data
Caspar Brekenfeld, MD	University Medical Center Hamburg Eppendorf, Department of Diagnostic and Interventional Neuroradiology, Hamburg, Germany	Site Investigator	Acquisition of data
Christopher E. J. Doppler, MD	University Hospital Cologne, Department of Neurology, Faculty of Medicine, University Cologne, Cologne, Germany	Site Investigator	Acquisition of data
Özgür A. Onur, MD	University Hospital Cologne, Department of Neurology, Faculty of Medicine, University Cologne, Cologne, Germany	Site Investigator	Acquisition of data
Christoph Kabbasch, MD	University Hospital Cologne, Department of Neuroradiology, Faculty of Medicine, University Cologne, Cologne, Germany	Site Investigator	Acquisition of data
