# Supplementary material for: Measuring Patient Safety Climate in Acute Stroke Therapy
Source: Front Neurol. 2021 Oct 1;12:686649. doi: 10.3389/fneur.2021.686649 (PMC8517443; doi:10.3389/fneur.2021.686649)
Supplement: Supplementary Table 2 — Items response rate, means, reliability characteristics and factor loading. [file Table_2.pdf]

Supplemental Table: Items response rate, means, reliability characteristics and factor loading

		Mean	SD	Missing (%)	Disagree (%)	Agree (%)	Corrected Item-Total Correlation	α if item deleted	Factor loading
	**Teamwork climate**								
1	Nurse input is well received in this clinical area.	3,80	0,85	0,6	6,7	71,2	0,57	0,87	0,59
2	In this clinical area, it is difficult to speak up if I perceive a problem with patient care.*	3,63	1,00	1,2	17,9	64,8	0,35	0,88	0,40
3	Disagreements in this clinical area are resolved appropriately (i.e., not who is right, but what is best for the patient)	3,48	0,90	0,6	17,2	57,7	0,60	0,87	0,67
4	I have the support I need from other personnel to care for patients.	3,91	0,80	0,6	6,1	79,8	0,60	0,87	0,69
5	It is easy for personnel in this clinical area to ask questions when there is something that they do not understand.	4,16	0,76	0,6	4,3	87,7	0,49	0,87	0,61
6	The physicians and nurses here work together as a well-coordinated team.	3,68	0,86	0,6	11,0	65,6	0,55	0,87	0,59
	**Safety climate**								
7	I would feel safe being treated here as a patient.	3,54	0,86	0,6	9,8	58,9	0,63	0,87	0,64
8	Medical errors are handled appropriately in this clinical area.	3,42	0,95	0,6	16,6	55,2	0,65	0,87	0,76
9	I know the proper channels to direct questions regarding patient safety in this clinical area.	3,80	0,95	0,6	12,3	68,1	0,58	0,87	0,68
10	I receive appropriate feedback about my performance.	3,15	1,02	1,2	28,4	40,7	0,57	0,87	0,63
11	In this clinical area, it is difficult to discuss errors.*	3,42	0,94	0,6	19,6	50,3	0,29	0,88	0,39
12	I am encouraged by my colleagues to report any patient safety concerns I may have.	3,35	0,84	1,2	13,6	42,6	0,45	0,87	0,52
13	The culture in this clinical area makes it easy to learn from the errors of others.	3,17	0,97	0,6	23,3	37,4	0,66	0,87	0,70
	**Job satisfaction**								
14	I like my job.	4,27	0,67	0,6	1,2	90,2	0,46	0,88	0,67
15	Working in this hospital is like being part of a large family.	3,99	0,92	0,6	8,6	77,9	0,53	0,87	0,57
16	This hospital is a good place to work.	3,90	0,84	0,6	10,5	76,5	0,66	0,87	0,87
17	I am proud to work at this hospital.	3,78	0,92	0,6	7,4	60,7	0,56	0,87	0,76
18	Moral in this clinical area is high.	3,89	0,91	0,6	8,0	73,0	0,51	0,87	0,58
	**Stress recognition**								
19	When my workload becomes excessive, my performance is impaired.	3,88	1,07	0,6	13,5	72,4	0,12	0,88	0,69
20	I am less effective at work when fatigued.	3,95	1,03	0,6	13,6	77,2	0,14	0,88	0,83
21	I am more likely to make errors in tense or hostile situations.	3,58	1,10	0,6	18,4	62,0	0,11	0,88	0,71
22	Fatigue impairs my performance during emergency situations.	3,63	1,08	2,4	15,0	63,8	0,02	0,88	0,83

Supplemental Table: Items response rate, means, reliability characteristics and factor loading

	**Perception of Management**								
	Unit level:								
23	Management supports my daily efforts.	3,60	1,03	0	14,6	62,2	0,57	0,87	0,65
24	Management does not knowingly compromise the safety of patients.	2,23	0,92	0	9,8	65,9	-0,49	0,89	excluded from analysis*
25	Problem personnel are dealt constructively in hospital.	3,22	0,95	0	22,0	40,2	0,54	0,87	0,72
26	I am provided with adequate, timely information about events in the hospital that might affect my work.	3,42	0,94	0	17,7	50,6	0,47	0,87	0,67
	Hospital level:								
27	Management supports my daily efforts:	2,67	1,09	0,6	41,7	22,1	0,41	0,88	0,58
28	Management does not knowingly compromise the safety of patients.	3,16	1,20	0,6	39,3	33,1	-0,23	0,89	excluded from analysis*
29	Problem personnel are dealt constructively in hospital.	2,86	0,94	0,6	28,2	19,0	0,49	0,87	0,66
30	I am provided with adequate, timely information about events in the hospital that might affect my work.	2,93	1,02	0	31,1	28,0	0,48	0,87	0,66
	**Working conditions**								
31	The levels of staffing in this clinical area are sufficient to handle the number of patients.	2,75	1,17	0	51,2	31,7	0,33	0,88	0,42
32	This hospital does a good job of training new personnel.	3,40	1,02	0	19,5	51,8	0,50	0,87	0,82
33	All the necessary information for diagnostic and therapeutic decisions is routinely available to me.	Excluded from questionnaire***							
34	Trainees in my discipline are adequately supervised.	3,41	0,98	0	19,5	56,1	0,60	0,87	0,89

Alpha (Cronbach's alpha) if item is deleted and factor loading regarding Confirmatory Factor analysis (CFA) are given.
* Items reverse scored.
** Item 24 and 28 show a strongly bimodal pattern, excluded from factor analysis.
*** Due to decision of interdisciplinary expert group item 33 of the German SAQ was not applicable to acute care of stroke patients and therefore excluded.
